# The CCHamide1 Neuropeptide Expressed in the Anterior Dorsal Neuron 1 Conveys a Circadian Signal to the Ventral Lateral Neurons in *Drosophila melanogaster*

**DOI:** 10.3389/fphys.2018.01276

**Published:** 2018-09-10

**Authors:** Yuri Fujiwara, Christiane Hermann-Luibl, Maki Katsura, Manabu Sekiguchi, Takanori Ida, Charlotte Helfrich-Förster, Taishi Yoshii

**Affiliations:** ^1^Graduate School of Natural Science and Technology, Okayama University, Okayama, Japan; ^2^Department of Neurobiology and Genetics, Theodor-Boveri Institute, Biocenter, University of Würzburg, Würzburg, Germany; ^3^Matching Program Course, Okayama University, Okayama, Japan; ^4^Division of Searching and Identification of Bioactive Peptides, Department of Bioactive Peptides, Frontier Science Research Center, University of Miyazaki, Miyazaki, Japan; ^5^Center for Animal Disease Control, University of Miyazaki, Miyazaki, Japan

**Keywords:** circadian clock, circadian rhythm, *Drosophila*, CCHamide1, pacemaker neuron, neuropeptide, pigment-dispersing factor

## Abstract

The fruit fly *Drosophila melanogaster* possesses approximately 150 brain clock neurons that control circadian behavioral rhythms. Even though individual clock neurons have self-sustaining oscillators, they interact and synchronize with each other through a network. However, little is known regarding the factors responsible for these network interactions. In this study, we investigated the role of CCHamide1 (CCHa1), a neuropeptide expressed in the anterior dorsal neuron 1 (DN_1a_), in intercellular communication of the clock neurons. We observed that CCHa1 connects the DN_1a_ clock neurons to the ventral lateral clock neurons (LN_v_) via the CCHa1 receptor, which is a homolog of the gastrin-releasing peptide receptor playing a role in circadian intercellular communications in mammals. *CCHa1* knockout or knockdown flies have a generally low activity level with a special reduction of morning activity. In addition, they exhibit advanced morning activity under light-dark cycles and delayed activity under constant dark conditions, which correlates with an advance/delay of PAR domain Protein 1 (PDP1) oscillations in the small-LN_v_ (s-LN_v_) neurons that control morning activity. The terminals of the s-LN_v_ neurons show rather high levels of Pigment-dispersing factor (PDF) in the evening, when PDF is low in control flies, suggesting that the knockdown of *CCHa1* leads to increased PDF release; PDF signals the other clock neurons and evidently increases the amplitude of their PDP1 cycling. A previous study showed that high-amplitude PDP1 cycling increases the siesta of the flies, and indeed, *CCHa1* knockout or knockdown flies exhibit a longer siesta than control flies. The DN_1a_ neurons are known to be receptive to PDF signaling from the s-LN_v_ neurons; thus, our results suggest that the DN_1a_ and s-LN_v_ clock neurons are reciprocally coupled via the neuropeptides CCHa1 and PDF, and this interaction fine-tunes the timing of activity and sleep.

## Introduction

It has been demonstrated that cyanobacteria, which are simple unicellular organisms, can generate robust circadian rhythms (Golden et al., 1997). In animals, however, multiple circadian pacemakers are required to form a network that coordinates behavioral rhythms. In mice, for example, the suprachiasmatic nucleus (SCN) in the anterior hypothalamus is the master clock, and is composed of approximately 20,000 pacemaker neurons (Welsh et al., 2010). Individual SCN oscillators contain autonomous transcriptional-translational feedback loops whereby the mRNA and proteins of clock genes are expressed in a circadian manner. The oscillators are synchronized and reinforced chemically using neurotransmitters such as γ-aminobutyric acid, gastrin-releasing peptide (GRP), and vasoactive intestinal polypeptide (VIP), thereby generating coherent and robust rhythms at the network level (Ramkisoensing and Meijer, 2015). The fruit fly *Drosophila melanogaster* has a similar but simpler circadian network in the brain (Beckwith and Ceriani, 2015; Hermann-Luibl and Helfrich-Förster, 2015). However, the detailed mechanisms of its connections are not fully understood.

Approximately 150 *Drosophila* clock neurons, which express clock genes and proteins in a circadian manner, can be divided into nine distinct clusters: four dorsal neuron clusters (DN_1a_ [anterior], DN_1p_ [posterior], DN_2_, and DN_3_), and five lateral neuron clusters (LPN, LN_d_, 5th s-LN_v_, l-LN_v_, and s-LN_v_). Most studies have concentrated on the role of the lateral neurons, among which the Pigment-dispersing factor (PDF)-expressing s-LN_v_ neurons appear to be the master pacemaker neurons in the circadian hierarchy (Helfrich-Förster, 1998; Renn et al., 1999; Grima et al., 2004; Shafer and Taghert, 2009; Yoshii et al., 2012). The PDF signaling from the s-LN_v_ neurons controls two PDF receptor (PDF-R)-positive LN_d_ neurons, and sets their free-running periods (Yao and Shafer, 2014; Liang et al., 2016). In contrast, some PDF-negative LNs, the 5th s-LN_v_ neuron and some LN_d_ neurons, act independently of PDF signaling or are weakly coupled to PDF-positive neurons. Despite the overwhelming importance of the s-LN_v_ neurons in *Drosophila*’s clock, recent studies indicate that the DNs play roles in the modulations of activity rhythms and sleep (Murad et al., 2007; Zhang et al., 2010; Kunst et al., 2014; Guo et al., 2016). Among the DNs, the DN_1a_ neurons are unique because they are functional from the early larval stages onward, and seem to interact with the s-LN_v_ neurons (Kaneko et al., 1997; Helfrich-Förster, 2003; Klarsfeld et al., 2004; Shafer et al., 2006). The DN_1a_ neurons express the PDF-R and respond to PDF by increasing their cyclic adenosine monophosphate (cAMP) levels (Shafer et al., 2008; Im and Taghert, 2010) and prolonging their circadian period (Yoshii et al., 2009b). On the other hand, DN_1a_ neurons express the Neuropeptide-like precursor 1-derived neuropeptide IPNamide (Shafer et al., 2006) and glutamate (together with a subset of the DN_1p_ neurons) (Hamasaka et al., 2007; Collins et al., 2012). Whereas the role of IPNamide is not known, glutamate signals s-LN_v_ neurons via the *Drosophila* metabotropic glutamate receptor DmGluRA (Hamasaka et al., 2007; Collins et al., 2012, 2014). Glutamate signaling decreases cAMP levels in the s-LN_v_ neurons and seems to shorten the circadian period of the flies (Hamasaka et al., 2007). Thus, there seems to be an interplay between DN_1a_ and s-LN_v_ neurons that is not yet well-understood.

Here, we focused on the DN_1a_ neurons and their role in the clock network. We observed that, in addition to IPNamide and glutamate, the DN_1a_ neurons express the neuropeptide CCHamide1 (CCHa1), which has been recently identified in *D. melanogaster* (Hansen et al., 2011; Ida et al., 2012). The LN_v_ neurons express the CCHa1 receptor (CCHa1-R), and the s-LN_v_ neurons respond to CCHa1 with an increase in cAMP levels, demonstrating CCHa1-dependent communication between the s-LN_v_ and DN_1a_ neurons. The loss of CCHa1 modifies the activity pattern of the flies, changes phase or amplitude of PDP1 oscillations in the different clock neurons, and alters PDF cycling in the s-LN_v_ terminals. Taken together, our study demonstrates that the CCHa1 neuropeptide plays a novel role as an intercellular communicator connecting the DN_1a_ and s-LN_v_ clock neurons.

## Materials and Methods

### Fly Strains

*Drosophila melanogaster w^1118^* (*w*; Bloomington Drosophila Stock Center [BDSC] #5905) flies were used as the control strain in this study. Additionally, the following strains were used: *w;tim(UAS)-Gal4* (*tim-Gal4*) (Blau and Young, 1999), *w;Pdf-Gal4* (Renn et al., 1999), *w;clk856-Gal4* (Gummadova et al., 2009), *y w;UAS-GFP S65T* (BDSC #1522), *w;UAS-Epac1-camps* (Shafer et al., 2008), *w*;*UAS-dicer2* (Vienna Drosophila Resource Center [VDRC] #60012), *w;UAS-CCHa1^RNAi^* (VDRC #104974), *w;UAS-CCHa1-R^RNAi^* (VDRC #103055), *Mi{MIC}CCHa1^MI09190^* (BDSC #51261), *Mi{MIC}^MI03750^* (BDSC #43865), *y w;CCHa1^SK8^* (Ren et al., 2015), and *daughterless-Gal4* (*da-Gal4*; BDSC #55851). To exclude unwanted mutations, *w;Pdf-Gal4, w;UAS-CCHa1^RNAi^*, *w;UAS-CCHa1-R^RNAi^*, *w;da-Gal4*, and *y w;CCHa1^SK8^* strains were outcrossed at least six times with *w* control flies. All RNAi strains and corresponding control strains co-expressed *UAS-dicer2* to enhance the RNAi efficiency (Dietzl et al., 2007). To generate the *CCHa1-R-Gal4* line, we employed the recombinase-mediated cassette exchange (RMCE) system to insert a *Gal4* construct into the 5′-untranslated region (UTR) of the *CCHa1-R* gene (Venken et al., 2011). The *Gal4* gene trap cassette, *pBS-KS-attB1-2-GT-SA-Gal4-Hsp70pA*, was injected into the *Mi{MIC}^MI03750^* line by BestGene (BestGene Inc., Chino Hills, CA, United States). The RMCE integration was verified by polymerase chain reaction (PCR), as described previously (Venken et al., 2011). Flies were reared at 25°C under a 12-h light: 12-h dark cycle (LD) on *Drosophila* medium (0.7% agar, 8.0% glucose, 3.3% yeast, 4.0% cornmeal, 2.5% wheat embryo, and 0.25% propionic acid).

### Activity Recording and Data Analysis

Male flies aged 3–6 days were used to record locomotor activity rhythms. Flies were confined to recording tubes containing agar/sugar food (2% agar and 4% sucrose) for the *Drosophila* Activity Monitor (DAM2, Trikinetics Inc., Waltham, MA, United States). The monitors were placed in an incubator (CN-40A, Mitsubishi Electric, Tokyo, Japan) and maintained at constant temperature of 20 (±0.25)°C. Standard cool white light-emitting diodes were set above the monitors in the incubator, and were controlled by an LC4 light controller (Trikinetics Inc.). The light intensity used in all experiments was 100 lux (3.2 μW⋅^-2^). We recorded the activity of flies in LD for 7 days, followed by 14 days of constant darkness (DD). Activity was recorded in 1-min bins using a conventional infrared light sensor that counted the number of beam crosses for each individual fly.

For visual inspection, raw data were displayed as actograms using ActogramJ^1^ (Schmid et al., 2011). Free-running periods in DD were determined using a chi-square periodogram analysis. If a robust peak above the 0.05 confidence level appeared in the periodogram, the period was designated as statistically significant (Sokolove and Bushell, 1978).

Average activity profiles and siesta durations were calculated on 3 consecutive days (days 5–7) in LD. The average activity profiles were smoothed using an 11-point moving average. To calculate siesta duration, daily sleep of individual flies was analyzed in 30-min intervals using a macro program written in Microsoft Excel (Microsoft, Redmond, WA, United States; Gmeiner et al., 2013). The times when flies began to sleep longer than 10 min in the morning and shorter than 10 min in the evening were assigned as the onset and offset of siesta, respectively. The average activity profiles in the 1st day of DD were smoothed using a 31-point moving average.

### Immunohistochemistry and Confocal Imaging

Whole flies were fixed in 4% paraformaldehyde in phosphate-buffered saline (PBS) with 0.1% Triton X-100 for 2.5 h at room temperature (RT). Fixed flies were washed three times with PBS, and then their brains were dissected. The brains were then washed three times with PBS containing 0.5% Triton X-100 (PBS-T), after which they were blocked in PBS-T containing 5% normal donkey serum for 1 h at RT and subsequently incubated with primary antibodies at 4°C for 48 h. After washing six times with PBS-T, the brains were incubated with secondary antibodies at RT for 3 h, washed six more times in PBS-T, and mounted in Vectashield mounting medium (Vector Laboratories, Burlingame, CA, United States). The primary antibodies used were rabbit anti-CCHa1 (Veenstra and Ida, 2014), chicken anti-green fluorescent protein (GFP) (1:1000; Rockland, Limerick, PA, United States), mouse anti-PDF (1:1000; Developmental Studies Hybridoma Bank) (Cyran et al., 2005), rabbit anti-PDP1 (1:9000) (Cyran et al., 2003), rat anti-TIMELESS (TIM) (1:3000) (Yoshii et al., 2008), and rabbit anti-*Gryllus* PDF (1:15000) (Abdelsalam et al., 2008). We used the following fluorescence-conjugated secondary antibodies at a 1:500 dilution: Alexa Fluor^®^ 488 nm (goat anti-chicken, goat anti-mouse), 555 nm (goat anti-rabbit), 647 nm (goat anti-mouse), antibodies (Life Technologies, Carlsbad, CA, United States); and goat anti-rabbit and anti-rat Cy3 antibodies (Millipore, Billerica, MA, United States).

Staining was visualized using laser scanning confocal microscopes (Olympus FV1200, Olympus, Tokyo, Japan). For quantification of immunostaining signal, the confocal microscope settings were kept consistent throughout the experiments. Measurement of staining intensity was performed using Fiji software (Schindelin et al., 2012), as described previously (Yoshii et al., 2009a). For quantification of PDP1 staining, the intensity values were obtained from all the stained clock neurons. In our confocal images, the size of the nuclei of clock neurons was approximately 8 pixels. Therefore, for each cell, a circle of 8 pixels marking the nucleus of the cell, where PDP1 is localized, was selected using the selection brush tool, and the mean intensity of these 8 pixels was measured. The background intensity was subtracted from the signals, and then the mean intensities per cell group and brain hemisphere were calculated. For quantification of CCHa1 staining, the polygon selection tool of Fiji was used to select each cell body of the DN_1a_ neurons. For quantification of PDF staining and area of branching at the s-LN_v_ terminals, the polygon selection tool was used to select the area of the branching s-LN_v_ dorsal terminals that were visualized upon Z-projection of multiple confocal stacks (Kozlov et al., 2017). To visualize projections of the DN_1a_, s-LN_v_, and l-LN_v_ neurons, we traced and reconstituted their projections using the Fiji plugin Simple Neurite Tracer (Longair et al., 2011).

### Live cAMP-Imaging

Male flies, aged 5–7 days, of the genotype *w;clk856-Gal4;UAS-Epac1-camps* or *UAS-dicer2;clk856-Gal4/CCHa1-R^RNAi^;UAS-Epac1-camps/+* were anesthetized on ice, and their brains were dissected in cold hemolymph-like saline (HL3) (Stewart et al., 1994). The experiments were conducted between zeitgeber time (ZT) 6–9. The brains were then mounted at the bottom of a plastic petri dish in HL3 with their anterior surface facing upward and allowed to recover from dissection for 10–15 min prior to imaging. An epifluorescence imaging setup (VisiChrome High Speed Polychromator System, ZEISS Axioskop2 FS plus, Visitron Systems GmbH, Puchheim, Germany) with a 40× water-immersion objective (ZEISS 40×/1.0 DIC VIS-IR) was used to conduct the experiments. The LN_v_ clock neurons were located according to their characteristic positions, and regions of interest were defined on single cell bodies using the VisiView Software (version 2.1.1, Visitron Systems GmbH). Time-lapse frames were acquired at 0.2 Hz for 750 s, exciting the cyan fluorescent protein (CFP) fluorophore of the ratiometric cAMP sensor with 405 nm light. Emissions of CFP and YFP were detected separately with a charge-coupled device camera (Photometrics, CoolSNAP HQ, Visitron Systems GmbH) using a beam splitter. After measuring baseline CFP and YFP levels for ∼100 s, substances were applied drop-wise using a pipette. The CCHa1 peptide (synthesized by Peptide Institute Inc., Osaka, Japan) was diluted in 0.1% dimethyl sulfoxide (DMSO) in HL3 to a concentration of 10^-5^ M; 10^-5^ M of the forskolin derivative, NKH^477^ in HL3 with 0.1% DMSO was used as positive control, whereas HL3 alone with 0.1% DMSO served as negative control. Inverse Förster resonance energy transfer (iFRET) was calculated according to the following equation: iFRET = CFP/(YFP-CFP^∗^0.357) (Shafer et al., 2008). In this equation, CFP and YFP are background-corrected raw fluorescence data, and 0.357 is subtracted from YFP fluorescence as this is the fraction of CFP spillover into the YFP channel in our imaging setup. Finally, iFRET traces of individual neurons were normalized to baseline levels and averaged for each treatment. To quantify and statistically compare the response amplitudes of each treatment, maximum iFRET changes were determined for individual neurons.

Statistics were performed using Systat (v 11.00.01, Systat Software Inc., San Jose, CA, United States). Data were compared using a one-way analysis of variance (ANOVA) test followed by *post hoc* pairwise comparison with Bonferroni correction. The *p*-values of data that were not normally distributed were corrected by multiplying by 2, according to Glaser (1978).

### Quantitative PCR

*UAS-dicer2;+/UAS-CCHa1-R^RNAi^* (control) and *UAS-dicer2;da-Gal4/UAS-CCHa1-R^RNAi^* strains were used to examine the efficiency of *CCHa1-R* RNAi; *da-Gal4* is ubiquitously expressed (Cronmiller and Cummings, 1993). Male flies were anesthetized on ice at ZT2 in LD, and 15 heads were quickly sampled for each strain. Total RNA was extracted using a spin column-based RNA extraction kit (RNA Basic Kit, Nippon Genetics, Tokyo, Japan); 120 ng of total RNA was used for reverse transcription (ReverTra Ace with gDNA Remover, Toyobo, Osaka, Japan). Quantitative PCR was performed by Mx3000P Real-Time PCR System (Agilent, CA, United States) using Thunderbird SYBR qPCR Mix (Toyobo, Osaka, Japan). We used the following primers: *CCHa1-R* forward primer, 5′-GTTTCCAGTCCCGTCACTCC-3′ and reverse primer, 5′-GTGCGTGTGCTTCATTGTGA-3′; *RpL13A* (used as a house-keeping gene) forward primer, 5′-AGCTGAACCTCTCGGGACAC-3′ and reverse primer, 5′-TGCCTCGGACTGCCTTGTAG-3′ (Ling and Salvaterra, 2011). The delta-delta CT method was used to determine the relative amount of the *CCHa1-R* mRNA.

## Results

### CCHa1 Is Expressed in the DN_1a_ Clock Neurons

**FIGURE 1:**
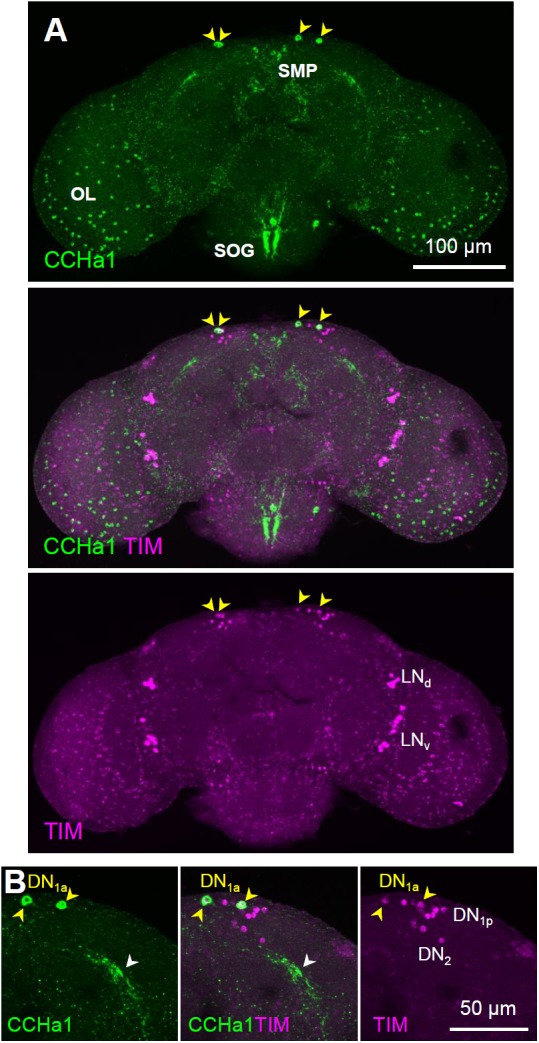
CCHa1 expression in the DN_1a_ clock neurons. **(A)** Double staining with anti-CCHa1 (green) and anti-TIM (magenta) antibodies revealed that only the DN_1a_ neurons (yellow arrowheads) express CCHa1; CCHa1 is also expressed in the entire optic lobe (OL) and in the cells around the superior medial protocerebrum (SMP) and suboesophageal ganglion (SOG). **(B)** An enlarged image of the DN_1a_ neurons. Yellow arrowheads and the white arrowhead indicate the cell bodies of the DN_1a_ neurons and their projections, respectively. CCHa1, CCHamide1; DN_1a_, anterior dorsal neuron 1; LN_d_, dorsal lateral neuron; LN_v_, ventral lateral neuron.

The CCHa1-immunoreactive neurons are widely distributed in the adult brain (**Figure 1A**). Many small cell bodies were stained in the optic lobes as well as relatively large cell bodies along the lateral protocerebrum, subesophageal ganglion, and intermediate and superior medial protocerebrum. To identify clock neurons, we performed a double staining with the anti-TIM and anti-CCHa1 antibodies; TIM and CCHa1 were clearly co-localized in the DN_1a_ neurons (**Figures 1A,B**) but not in other clock neurons (**Supplementary Figure S1**).

### Rhythmic Expression of CCHa1 in the DN_1a_

**FIGURE 2:**
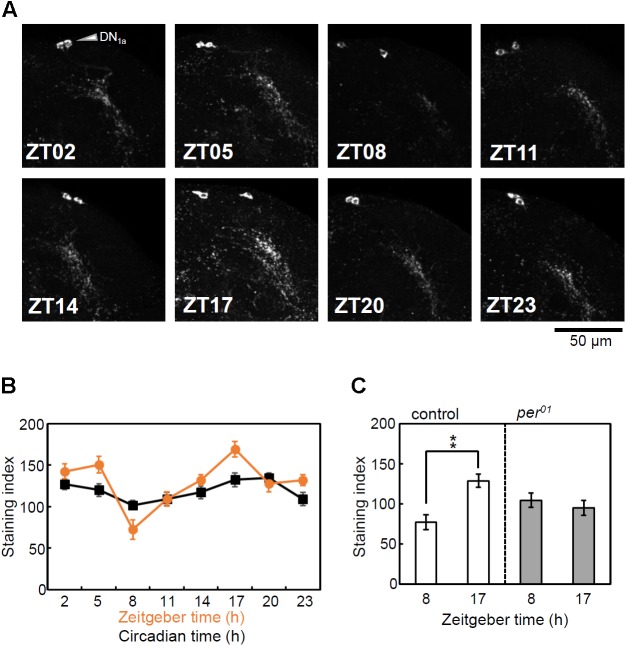
Rhythmic expression of CCHa1. **(A)** Immunostaining using anti-CCHa1 antibody of the DN_1a_ neurons at 3 h intervals during LD in *w* control flies. **(B)** Mean CCHa1 staining intensity (±SEM) of the cell bodies in LD (orange) and DD (black). The LD data represent pooled results from two independent experiments, and DD data represent pool results from three independent experiments. Hemispheres of nine different brains were analyzed for each time point in a single experiment (resulting in *n* = 18 for the LD experiment and *n* = 27 for the DD experiment). CircWave analysis (version 1.4, Dr. R. Hut, http://www.euclock.org) revealed that CCHa1 is expressed in a circadian manner in LD (*p* < 0.01, *n* = 18) and DD (DD, *p* < 0.05, *n* = 27). Similarly, a one-way analysis of variance (ANOVA) revealed that staining intensity is significantly dependent on time in LD [*F*_(7)_ = 9.565, *p* < 0.01] and DD [*F*_(7)_ = 2.576, *p* < 0.05]. **(C)** Effect of the *per*^01^ mutation on cyclic CCHa1 expression. The *w* control flies showed a significant difference between ZT8 and ZT17 [Kolmogorov–Smirnov test, followed by *t*-test, *t*_(16)_ = 3.953, ^∗∗^*p* < 0.01, *n* = 9]. In contrast, the *per^01^* mutants did not show a significant difference [Kolmogorov–Smirnov test, followed by *t*-test, *t_(_*_16)_ = –0.709, *p* > 0.05, *n* = 9].

Circadian expressions of PDF in the s-LN_v_ and the Ion transport peptide (ITP) in the 5th s-LN_v_ and ITP-positive LN_d_ have been previously reported (Park et al., 2000; Hermann-Luibl et al., 2014). Therefore, we investigated whether the expression of CCHa1 in the DN_1a_ neurons is also regulated by the circadian clock. While the rhythmic changes in PDF and ITP levels are observed in their axonal projections, obvious difference in CCHa1 staining intensity was observed not only in the DN_1a_ terminals but also in the cell bodies (**Figure 2A**). Since the diffused and fine dendritic arborizations of the DN_1a_ terminals made an objective quantification difficult, we quantified CCHa1 staining only in the cell bodies. We did so for two independent experiments in LD and three independent experiments in DD. CircWave (Oster et al., 2006) and one-way ANOVA revealed circadian changes in staining intensity in DN_1a_ cell bodies in all experiments except for one in DD (**Supplementary Figure S2**). When we pooled the two experiments in LD and three experiments in DD, CircWave and ANOVA revealed circadian rhythms under both conditions (LD and DD) (**Figure 2B**). In LD, CCHa1 exhibits a trough at ZT8 and a peak at ZT17 (**Figure 2B** and **Supplementary Figure S2**). In DD, the rhythm maintains a similar phase, but with dampened amplitude.

To investigate the effect of the clock on the CCHa1 rhythm, the brains of *per^01^* mutants together with control flies were immunostained at two time points, ZT8 and ZT17, in LD. The CCHa1 staining intensity was significantly different between the two time points in control flies, whereas *per^01^* mutants did not show any difference (**Figure 2C**), suggesting that the rhythmic expression of CCHa1 is controlled by the clock.

### The CCHa1 Receptor Gene Is Expressed in the Ventrolateral Clock Neurons

**FIGURE 3:**
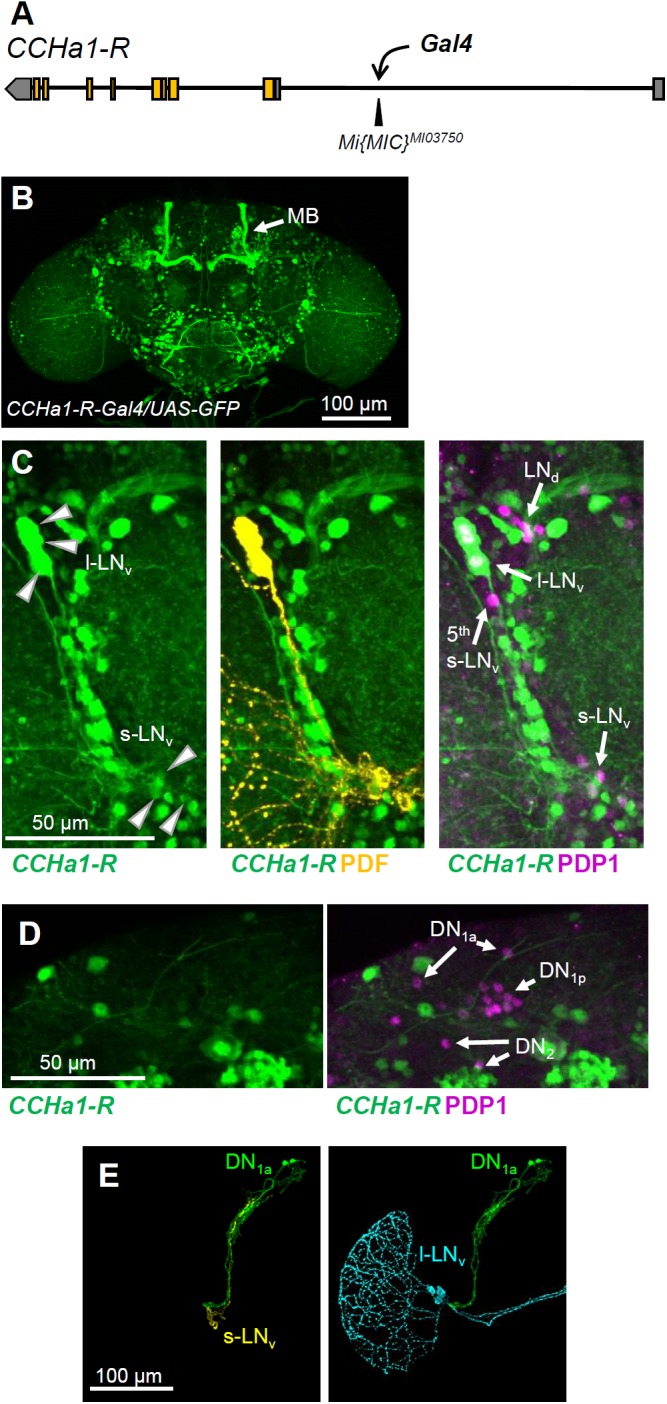
Expression pattern of *CCHa1-R* in the adult brain. **(A)** The *Gal4* construct was inserted into the 5′-untranslated region of the *CCHa1-R* gene in the *Mi{MIC}^MI03750^* strain. **(B)** Immunostaining of the *CCHa1-R-Gal4/UAS-GFP* strain using an anti-GFP antibody revealed that *CCHa1-R* is widely distributed in the adult brain, including mushroom bodies (MB, arrow). **(C)** Triple immunostaining using anti-GFP (green), anti-PDF (yellow) and anti-PDP1 (magenta) antibodies further revealed that *CCHa1-R* is expressed weakly in the s-LN_v_ and strongly in l-LN_v_ neurons. **(D)** The DN groups do not express *CCHa1-R.* **(E)** Projection patterns of the DN_1a_ and PDF-positive neurons; the *Mi{MIC}CCHa1^MI09190^* line, in which the *gfp* gene trap cassette was inserted into the second exon of the *CCHa1* gene, was used to visualize the projections of the DN_1a_ neurons. The projections of the DN_1a_ (green), s-LN_v_ (yellow), and l-LN_v_ neurons (light blue) were manually traced and reconstituted using the Fiji plugin Simple Neurite Tracer.

To investigate the CCHa1-R localization in the *Drosophila* brain, we generated a *CCHa1-R-Gal4* line using the RMCE method in the *Mi{MIC}^MI03750^* line. For this, the *Minos*-mediated integration cassette was inserted using site-directed mutagenesis in the 5′-UTR intron of the *CCHa1-R* gene (**Figure 3A**) (Venken et al., 2011). The *CCHa1-R-Gal4/UAS-GFP* line showed GFP expression in the entire brain, including strong expression in the mushroom bodies (**Figure 3B**). Furthermore, triple staining with anti-GFP, anti-PDP1, and anti-PDF antibodies revealed that *CCHa1-R* is expressed in the l-LN_v_ and s-LN_v_ neurons but not in other clock neurons (**Figures 3C,D**). Moreover, GFP was strongly expressed in the l-LN_v_ neurons and weakly in the s-LN_v_ neurons, suggesting that the levels of the *CCHa1-R* expression are different between these cell groups.

Using the *Mi{MIC}CCHa1^MI09190^* line, in which the *gfp* gene trap cassette was inserted into the second exon of the *CCHa1* gene, we were able to clearly visualize that the DN_1a_ projections run along the s-LN_v_ dorsal projections (**Figure 3E**), in agreement with previous studies (Shafer et al., 2006; Helfrich-Förster et al., 2007).

### The s-LN_v_ Neurons Respond to CCHa1 With an Increase in cAMP Levels

**FIGURE 4:**
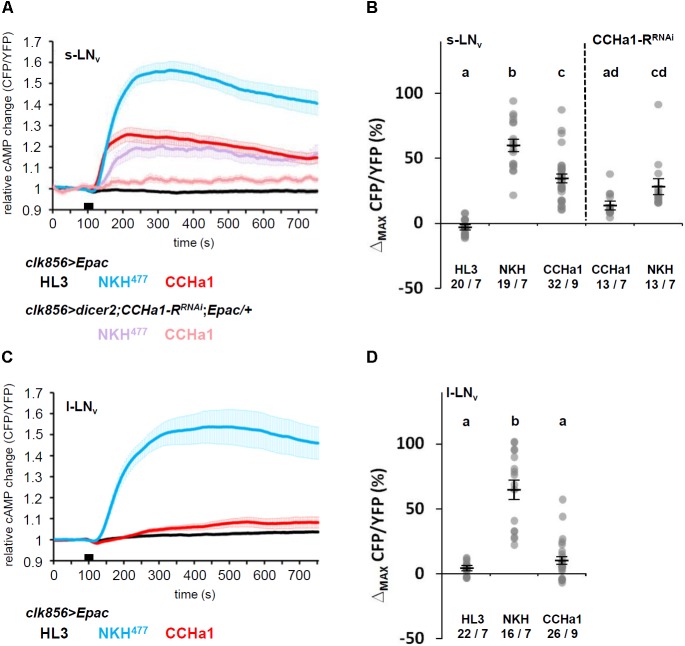
*Ex vivo* live cAMP-imaging upon CCHa1 application to PDF-neurons. **(A,C)** Mean iFRET traces of s-LN_v_ **(A)** and l-LN_v_ **(C)** neurons over 750 s reflect intracellular changes in cAMP; application time is indicated by the black bar. The experiments were conducted between ZT6–9. Application of hemolymph-like saline (HL3, black) and NKH^477^ (blue or purple) served as negative and positive controls, respectively. The cAMP level was increased only in s-LN_v_ but not l-LN_v_ neurons in response to bath-application of the CCHa1 peptide (red). This response was reduced in the s-LN_v_ neurons when *CCHa1-R* was knocked down by RNAi (rosy). Error bars represent SEM. **(B,D)** Quantification of maximal inverse FRET changes for each treatment in the s-LN_v_ **(B)** and l-LN_v_ **(D)** neurons; changes after NKH^477^-application were significantly different from the negative control in both neuronal subgroups (*p* < 0.001), whereas changes after CCHa1-application were different from the negative control only in the s-LN_v_ neurons (*p* < 0.001). When *CCHa1-R* was knocked down, s-LN_v_ responses to CCHa1 were not significantly different from the negative HL3 control (*p* > 0.05), whereas application of NKH^477^ still evoked significant increases in cAMP levels (*p* < 0.001). There was, however, no difference between CCHa1 and NKH^477^ applications in the *CCHa1-R* knockdown. Gray dots represent values of single neurons, black horizontal lines indicate mean ± SEM. Statistical significances are indicated by the letter code (values that were not statistical different from each other are marked by the same letter). Numbers at the bottom indicate numbers of neurons/numbers of brains.

The receptor of CCHa1 is a G protein-coupled receptor and belongs to the bombesin receptor subtype 3 (Hewes and Taghert, 2001; Ida et al., 2012). To investigate the physiological response to CCHa1 in the s-LN_v_ and l-LN_v_ neurons, we conducted live cAMP imaging in the adult brain using a *UAS* line expressing a genetically encoded cAMP FRET sensor, *UAS-Epac1-camps* (Nikolaev et al., 2004; Shafer et al., 2008). To evaluate our imaging method, we recorded inverse FRET values (CFP/YFP ratios) for single s-LN_v_ and l-LN_v_ cell bodies in living brains of *clk856-Gal4;UAS-Epac1-camps* flies. The flies were treated with HL3 saline or NKH^477^ (an adenylyl cyclase activator) which acted as negative and positive controls, respectively. Both the s-LN_v_ and l-LN_v_ neurons responded to bath application of 10^-5^ M NKH^477^ by increasing cAMP, and did not respond to HL3 (**Figure 4**). Interestingly, the s-LN_v_ neurons showed a significant increase in cAMP levels in response to 10^-5^ M CCHa1, whereas the l-LN_v_ neurons did not. To determine whether the s-LN_v_ neurons directly respond to CCHa1 through CCHa1-R, we knocked down *CCHa1-R* in the s-LN_v_ neurons (*dicer2;clk856-Gal4/UAS-CCHa1-R^RNAi^;UAS-Epac1-camps/+*), and repeated cAMP imaging. The efficiency of *CCHa1-R* knockdown was verified by quantitative PCR using *da-Gal4* (**Supplementary Figure S3A**). Since we used a single copy of *UAS-Epac1-camps* in this imaging experiment, the inversed FRET values in response to the application of NKH^477^ were lower than in the *clk856-Gal4;UAS-Epac1-camps* flies. Nevertheless, the response to CCHa1 was severely reduced after knockdown of its receptor (**Figures 4A,B**).

### Effect of CCHa1 on Activity Rhythms

**FIGURE 5:**
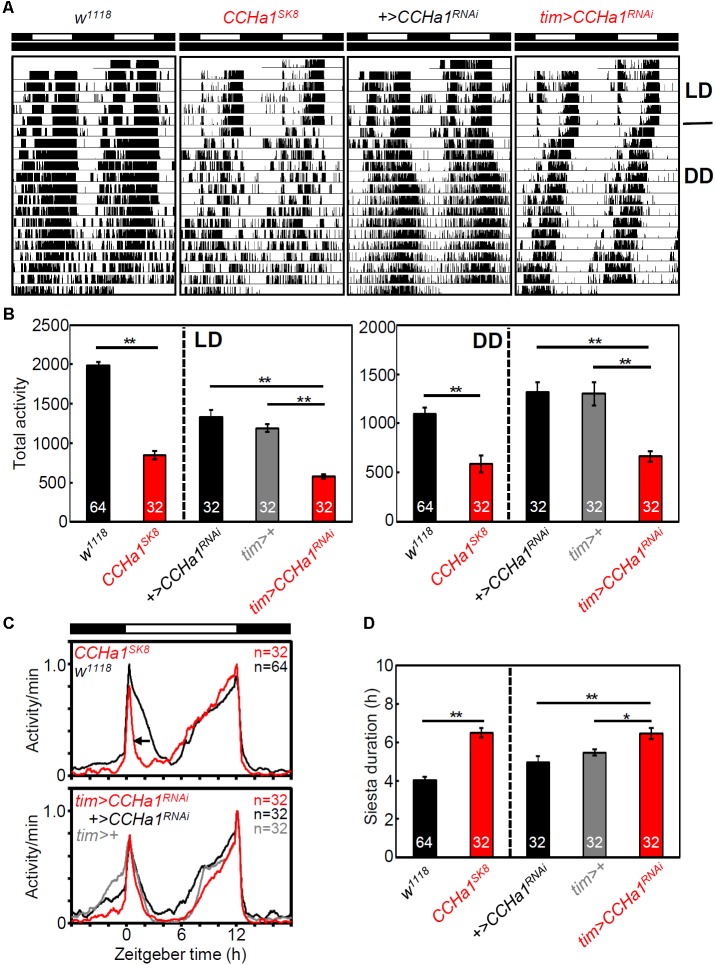
CCHa1 affects morning activity peak and total activity level. **(A)** Representative actograms of *w* control, *CCHa1* mutant (*CCHa1^SK8^*), *UAS* control (*+>CCHa1^RNAi^*), and *CCHa1* knockdown (*tim > CCHa1^RNAi^*) strains. **(B)** Mean total activity levels (±SEM) in LD and DD. **(C)** Average activity profiles of *CCHa1* mutants and *CCHa1* knockdown flies. The daily average activity for each strain was calculated from the last 3 days of LD. All activity profiles were normalized to 1. **(D)** Mean siesta durations (±SEM) of *CCHa1* mutants and *CCHa1* knockdown flies. Numbers in the columns indicate the number of flies. One-way ANOVA with Tukey’s multiple comparison test after the Kolmogorov–Smirnov test; ^∗^*p* < 0.05, ^∗∗^*p* < 0.01.

**FIGURE 6:**
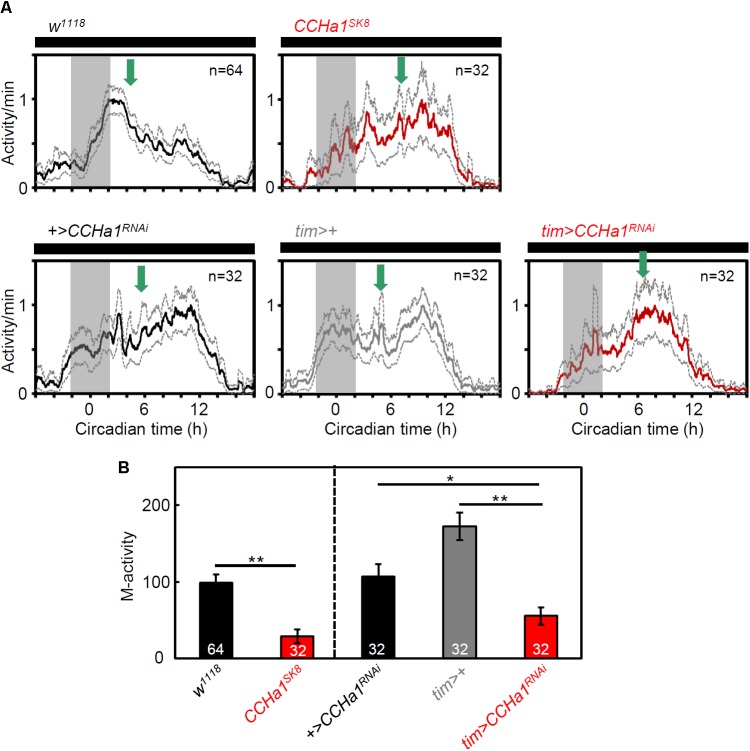
Morning activity of *CCHa1* mutants and *CCHa1* knockdown flies on the 1st day in DD. **(A)** Average activity profiles (±SEM) of *w^1118^* control flies, *CCHa1^SK8^* mutants, *UAS* control flies (*UAS-dicer2;+/UAS-CCHa1^RNAi^* = *+>CCHa1^RNAi^*), *tim-Gal4* control flies (*UAS-dicer2;tim-Gal4/+* = *tim>+*), and *CCHa1* knockdown flies (*UAS-dicer2;tim-Gal4/UAS-CCHa1^RNAi^* = *tim > CCHa1^RNAi^*). All activity profiles were normalized to 1. Green arrows point to the mean acrophase in the different strains calculated by ActogramJ. Compared with *w^1118^*, *+>CCHa1^RNAi^*, and *tim>+* flies, the acrophases of *CCHa1* mutants and *CCHa1* knockdown flies were phase-delayed. **(B)** Activity (mean ± SEM) of *CCHa1* mutants and *CCHa1* knockdown flies in the morning phase (gray area shown in **A**). Kruskal–Wallis test followed by Bonferroni correction; ^∗^*p* < 0.05, ^∗∗^*p* < 0.01.

To investigate the effect of CCHa1 on behavioral rhythms, we recorded locomotor activity rhythms of *CCHa1* mutants and *CCHa1* knockdown flies under LD and DD. The efficiency of *CCHa1* knockdown was verified by immunostaining (**Supplementary Figure S3B**). *CCHa1* mutants and *CCHa1* knockdown flies were significantly less active than the corresponding control flies (**Figures 5A,B**); this mainly concerned morning activity. In LD, the morning activity bout was narrower in flies without CCHa1, and in case of the mutants, the offset of morning activity was much earlier, which resulted in a longer siesta (**Figures 5C,D**). In the *CCHa1* knockdown flies, this was less evident, but they still slept significantly longer during their siesta than the controls (**Figure 5C**). Moreover, under DD, *CCHa1* mutants and *CCHa1* knockdown flies were significantly less active in the morning than the corresponding control flies, resulting in a shift of most activities into the second half of the active phase (**Figures 6A,B**). Altogether, our results suggest that CCHa1 is particularly involved in the generation of robust morning activity and in its timing. Under LD, the diminished morning activity of flies lacking CCHa1 appeared advanced, whereas under DD it appeared delayed.

Most interestingly, the absence of CCHa1 had virtually no influence on the free-running period in DD. Although *tim > CCHa1-R^RNAi^* and *clk856 > CCHa1^RNAi^* flies showed slightly but significantly shorter free-running periods than the corresponding *UAS* and *Gal4* control strains (**Table 1**), the free-running periods of *CCHa1^SK8^* null mutants and *tim > CCHa1^RNA^*^i^ flies were not significantly different from their control strains. Thus, even if there is a difference in the free-running period, it would be small, and the genetic background may influence the period more than CCHa1.

**Table 1:** Free-running activity rhythms in DD.

Genotype	*n*	Period (h) ± SEM	Power ± SEM	*R* (%)
*w^1118^*	62	24.2 ± 0.1	151.6 ± 6.6	90.3
*CCHa1^SK8^*	32	24.2 ± 0.1	178.3 ± 8.5^∗^	100
*CCHa1^MI09190^*	31	23.7 ± 0.1^∗∗^	123.9 ± 7.8^∗^	90.3
*tim/+*	32	24.6 ± 0.1	214.5 ± 8.6	100
*clk856/+*	31	24.1 ± 0.1	143.7 ± 8.6	83.9
*+/CCHa1^RNAi^*	32	24.5 ± 0.1	211.4 ± 8.7	96.9
*+/CCHa1-R^RNAi^*	32	24.5 ± 0.1	153.5 ± 6.7	96.9
*tim > CCHa1^RNAi^*	32	24.2 ± 0.1	175.8 ± 11.7^∗^	96.9
*clk856 > CCHa1^RNAi^*	63	23.8 ± 0.1^∗∗^	151.4 ± 5.7	95.2
*tim > CCHa1-R^RNAi^*	32	23.5 ± 0.1^∗∗^	173.4 ± 9.2	96.9

*DD, constant darkness; CCHa1, CCHamide1; SEM, standard error of the mean. The free-running period, power, and rhythmicity (R) was determined from data of days 1–10 of DD using χ^2^-periodogram analysis. *CCHa1* mutants and RNAi strains were compared with the corresponding control lines, e.g., *UAS* or Gal4 control lines. If period or power was significantly different from both of the corresponding control lines, it was designated as statistically significant. One-way ANOVA with Tukey’s multiple comparison test was used after testing for the normal distribution of data using the Kolmogorov–Smirnov test; ^∗^p < 0.05, ^∗∗^p < 0.01*.

### Effect of CCHa1 on Molecular Oscillations in Clock Neurons

**FIGURE 7:**
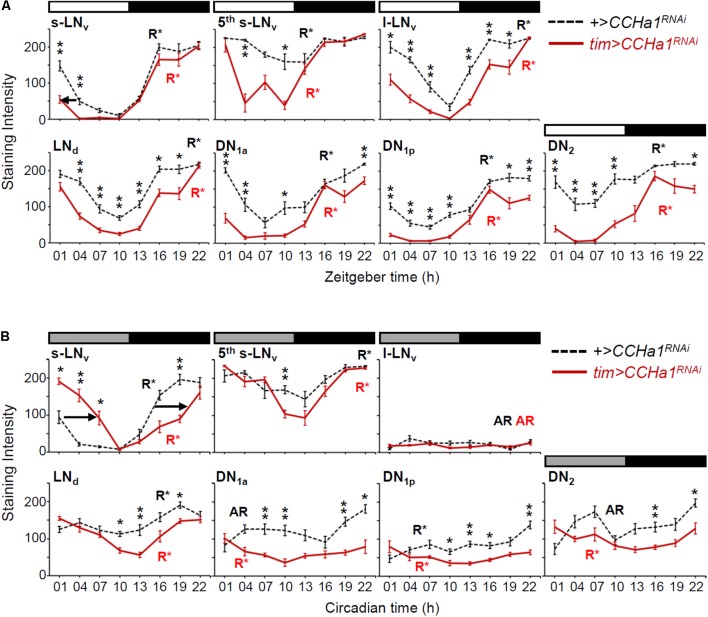
Cyclic expression of the PDP1 clock protein in the *CCHa1* knockdown flies. The PDP1 levels (mean ± SEM) in clock neurons were measured at 3-h intervals during LD **(A)** and DD **(B)**. Circadian time in DD does not indicate the exact subjective time but simply indicates the original zeitgeber time. Hemispheres of 9 different brains were analyzed for each time point. Dashed and colored solid lines indicate the data of the control (*UAS-dicer2;+/UAS-CCHa1^RNAi^*) and the RNAi (*UAS-dicer2;tim-Gal4/UAS-CCHa1^RNAi^*) strains, respectively. The rhythmicity of PDP1 expression is indicated by R^∗^ (rhythmic) or AR (arrhythmic), analyzed by CircWave (*p* < 0.01). **(A)** The decrease in PDP1 levels in the morning was slightly phase-advanced in the s-LN_v_ neurons (arrow). **(B)** The PDP1 cycling in the RNAi flies was significantly phase-delayed in the s-LN_v_ neurons compared with that of the control flies (arrows). We performed Mann–Whitney *U* test followed by Bonferroni correction to examine the effect of *CCHa1* knockdown for each time point (^∗^*p* < 0.05, ^∗∗^*p* < 0.01).

Since CCHa1 signaling from the DN_1a_ neurons mainly affects morning activity of the flies, it should influence the molecular oscillations of the s-LN_v_ neurons that control it. To test this, we measured the abundance of the PDP1 clock protein in clock neurons of *CCHa1* knockdown flies during LD and DD (**Figure 7**). We sampled brains at day 5 (starting from circadian time [CT] 1) in DD, because we expected an internal desynchronization among the clock neurons in *CCHa1* knockdown flies after several cycles of a free-running condition but not merely after the 1st day of DD. As expected, the largest differences in PDP1 oscillations between *tim > CCHa1^RNAi^* and control flies were present in the s-LN_v_ neurons. In LD, the *CCHa1* knockdown phase-advanced the decrease of PDP1 in the morning (arrow in **Figure 7A**), which fits the earlier decrease in morning activity that we observed. In DD, it phase-delayed the entire PDP1 oscillation (arrows in **Figure 7B**), which correlates with the delay in the activity of the mass center (green arrows in **Figure 6**).

The *CCHa1* knockdown also affected the other clock neurons significantly. It reduced PDP1 levels in most neurons. In LD, this reduction occurred predominantly during the time at which the PDP1 level of controls was already at its trough. Consequently, the knockdown enhanced the amplitudes of the PDP1 oscillations (**Figure 7A**). In DD, a higher-amplitude cycling of PDP1 after *CCHa1* knockdown occurred only in the 5th s-LN_v_ and LN_d_ neurons (**Figure 7B**). In the other neurons, the PDP1 oscillations were very weak or even absent already in the control flies. Especially, the l-LN_v_ neurons showed very low levels of PDP1 in both control and *tim > CCHa1^RNAi^* flies (**Figure 7B**), consistent with a previous report indicating that molecular oscillations in the l-LN_v_ neurons require light input via Cryptochrome (Yoshii et al., 2015). The DN_1a_, DN_1p_, and DN_2_ neurons showed very weak PDP1 oscillations peaking at approximately CT22 in control flies, but they were almost arrhythmic in *tim > CCHa1^RNAi^* flies. Thus, these results suggest that CCHa1 signaling has an impact on molecular oscillations and particularly modulates the phase of the s-LN_v_ neurons.

### Effect of CCHa1 on PDF Levels

**FIGURE 8:**
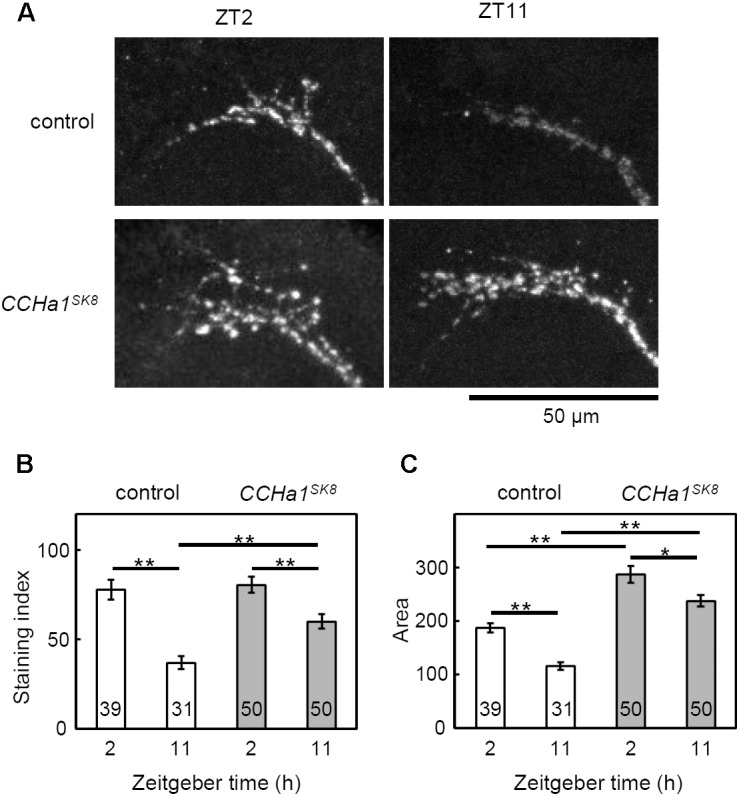
PDF-immunoreactivity of *CCHa1* mutants in the s-LN_v_ terminals. **(A)** Representative images of PDF immunostaining in the s-LN_v_ dorsal protocerebrum terminals. The samples were collected at ZT2 and ZT11. The upper panels indicate results of PDF staining from the *w^1118^* control strain and the lower panels indicate the same from *CCHa1^SK8^* mutants. **(B)** The PDF staining intensity was measured at the s-LN_v_ terminals. **(C)** Data show mean intensity (±SEM) calculated from results pooled from three independent experiments. Numbers in the columns indicate the number of brain hemispheres used. One-way ANOVA with Tukey’s multiple comparison test after the Kolmogorov–Smirnov test; ^∗^*p* < 0.05, ^∗∗^*p* < 0.01.

Since the small morning activity peak in *CCHa1* mutants in LD is reminiscent of *Pdf^01^* mutants (Renn et al., 1999), we investigated the effect of *CCHa1* on PDF levels. The s-LN_v_ terminals at the dorsal protocerebrum show circadian changes in PDF level and their axonal morphology, by which the time information appears to be conveyed from the s-LN_v_ neurons to other clock neurons in a time-dependent manner (Park et al., 2000; Fernandez et al., 2008). The *w* control flies showed significant differences between ZT2 and ZT11 in PDF staining intensity and the area of the axonal arborizations of the s-LN_v_ terminals (**Figure 8**), in agreement with a previous study (Fernandez et al., 2008). While *CCHa1* null mutants also showed a difference in PDF level, their PDF level at ZT11 was higher than that of control flies, leading to a lower amplitude of PDF cycling in the *CCHa1* mutants (**Figure 8B**). The same tendency was also observed in the area of the s-LN_v_ axonal arborizations (**Figure 8C**). At both time-points, the arborization pattern of the s-LN_v_ terminals in *CCHa1* mutants was more extensive than that in control flies. Therefore, the temporal morphological changes in the axonal arborizations were less clear in the mutants. This indicates an effect of CCHa1 on the PDF-dependent output from the s-LN_v_ neurons.

## Discussion

### CCHa1 Is a New Communication Factor in the Circadian Clock Network

**FIGURE 9:**
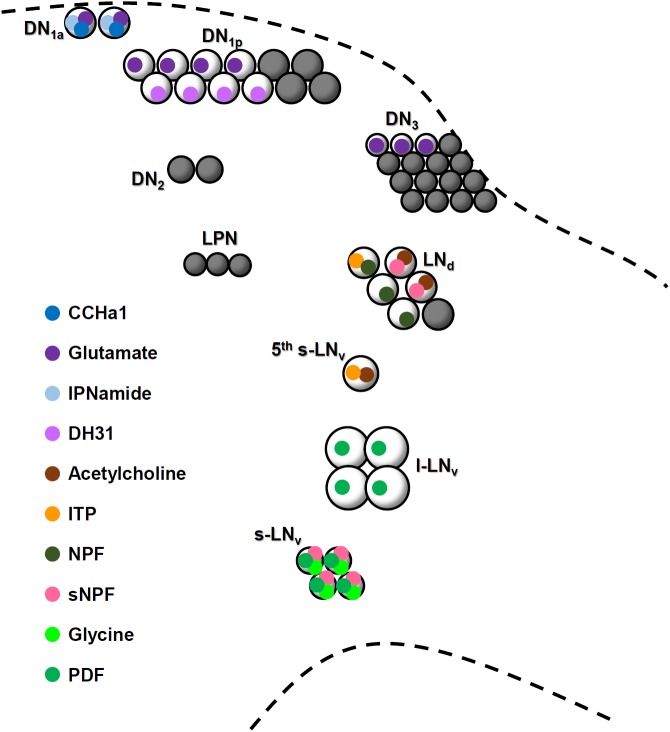
A schematic model showing neurotransmitter content in clock neuron clusters. Circles representing neurotransmitters that are filled with black have not been identified yet. Glutamate and DH31 are expressed in the DN_1p_ neurons but it is not known whether they are co-localized.

Central circadian clocks in animals are composed of multiple pacemaker clock neurons that utilize different “neuromessengers” (neuropeptides and classical neurotransmitters) (**Figure 9**) (Muraro et al., 2013; Beckwith and Ceriani, 2015; Hermann-Luibl and Helfrich-Förster, 2015). Supposedly, these pacemaker neurons communicate with each other to generate coherent and robust circadian rhythms. However, little is known as to how these clock neurons are interconnected, even in well-studied model organisms such as the mouse and fruit fly. Here, we show that the DN_1a_ and s-LN_v_ neurons of the adult fly are closely connected via neurites, and that they communicate with each other. We describe CCHa1 as novel circadian neuropeptide that is expressed in the DN_1a_ and signals the s-LN_v_ neurons. So far, it was known that the DN_1a_ neurons express IPNamide and glutamate (Shafer et al., 2006; Hamasaka et al., 2007; Collins et al., 2014) whereas the s-LN_v_ neurons express PDF, short neuropeptide F (sNPF), and glycine (Helfrich-Förster, 1995; Johard et al., 2009; Frenkel et al., 2017) (**Figure 9**). Thus, together with CCHa1, both types of clock neurons utilize two neuropeptides and one classical transmitter—and they are so far the only *Drosophila* clock neurons with three neuromessengers (**Figure 9**). While it is unclear whether IPNamide and sNPF mediate signals within the clock network (at least sNPF appears to signal downstream neurons (Selcho et al., 2017)), glutamate, PDF, and glycine participate in communication between the s-LN_v_ and DN_1a_ neurons (Hamasaka et al., 2007; Shafer et al., 2008; Collins et al., 2014; Frenkel et al., 2017). Glutamate signals originating from the DN_1a_ neurons (and other DN neurons) are transmitted via inhibitory metabotropic glutamate receptors to the s-LN_v_ neurons, and reduce their cAMP and Ca^2+^ levels (Hamasaka et al., 2007; Collins et al., 2014). Glycine also exerts inhibitory effects via ionotropic Cl^-^ channels but the signals are transmitted from the s-LN_v_ to the DN_1a_ neurons, where it reduces spiking frequencies (Frenkel et al., 2017). On the other hand, PDF increases cAMP levels in the DN_1a_ neurons (Shafer et al., 2008). Similar to PDF, CCHa1 appears to exert excitatory effects, but in the opposite direction: it is secreted from the DN_1a_ neurons and increases cAMP levels in the s-LN_v_ neurons. In summary, the DN_1a_ and s-LN_v_ neurons appear to be mutually connected via excitatory and inhibitory neurotransmitters, respectively.

Regarding CCHa1, we detected the expression of its receptor not only on the s-LN_v_, but also on the l-LN_v_ neurons. Nevertheless, only the s-LN_v_ neurons responded to the application of CCHa1 with an increase in cAMP levels. This does not imply that CCHa1 does not exert any effect on the l-LN_v_ neurons. There are several possibilities that can explain our results. Either the l-LN_v_ neurons are less responsive to CCHa1 and would need a higher concentration to respond, or activation of the CCHa1 receptor induces a different signaling cascade that leads to an increase in Ca^2+^ levels instead of cAMP. Since we used a rather high concentration of CCHa1 (10^-5^ M), we believe that the second possibility is more likely. For example, PDF signals not only through cAMP, but also weakly through Ca^2+^ (Mertens et al., 2005). In the cockroach, PDF increases cAMP levels in some clock neurons and Ca^2+^ levels in others (Wei et al., 2014; Gestrich et al., 2018). Thus, it will be interesting to investigate whether the l-LN_v_ neurons respond to CCHa1 with an increase in Ca^2+^ levels (see also the paragraph “comparison with the mammalian system”).

### The Role of CCHa1 in the Clock Network

The level of CCHa1 in the DN_1a_ neurons is low in the evening and high in the night-morning (**Figure 2**). Similarly, a recent study reported that *CCHa1-R* mRNA expression in the LN_v_ neurons is high in late night-morning and low in the evening (Abruzzi et al., 2017), suggesting that the s-LN_v_ neurons receive CCHa1 signals in a circadian manner. Perhaps most of the CCHa1 is secreted in the morning, a time at which the s-LN_v_ neurons are thought to secrete PDF (Collins et al., 2014; Liang et al., 2017). Thus, the DN_1a_ neurons also receive PDF signals in the morning, implying that the DN_1a_ and s-LN_v_ neurons are reciprocally and temporally coupled. This might explain why morning activity is not only strongly reduced when the s-LN_v_ lack PDF, but also when CCHa1 signaling is absent (**Figure 5**).

The CCHa1 signals from the DN_1a_ neurons seem to be important for a normal rhythmic release of PDF, as can be judged from the flattened amplitude of PDF cycling and daily s-LN_v_ terminal remodeling in *CCHa1^SK8^* mutants (**Figure 8**). As the latter finding is based on the observation of PDF immunostaining but not GFP staining, as in previous studies (Fernandez et al., 2008; Depetris-Chauvin et al., 2014; Petsakou et al., 2015), we might have overlooked smaller morphological changes. Nevertheless, the high PDF staining in *CCHa1* mutants revealed a complex arborization pattern of the s-LN_v_ terminals at the two tested time-points. Furthermore, it has been shown that the level of PDF is responsible for the daily remodeling of the s-LN_v_ terminals (Depetris-Chauvin et al., 2014). Thus, CCHa1 signaling may modulate the level of PDF, resulting in suppression of the daily remodeling of the s-LN_v_ terminals in *CCHa1* mutants. It is not clear whether the high level of PDF in the s-LN_v_ terminals indicates a high PDF release throughout day and night, but it is imaginable that a permanent PDF release exerts similar negative effects on the normal expression of morning activity as does an excessively low release of PDF. Additionally, the changed modulation of PDF signaling by CCHa1 would indirectly affect the other clock neurons that express the PDF receptor. Indeed the PDP1 oscillations in *CCHa1* knockdown flies were changed: under LD, they showed a high-amplitude PDP1 cycling in all clock neurons, and under DD, PDP1 cycling was enhanced in the 5th s-LN_v_ and LN_d_ neurons, but dampened in the remaining clock neurons (**Figure 7**). An earlier study showed that the amplitude of PDP1 cycling is an indicator of the siesta length in LD (Kistenpfennig et al., 2018). Mutants that lacked Cryptochrome (*cry^01^* mutants) exhibited high-amplitude PDP1 cycling and a long siesta, identical to our findings in the *CCHa1* mutants and *CCHa1* knockdown flies (**Figure 7**). In DD, the situation is apparently more complicated since only the LNs showed high-amplitude cycling. Nevertheless, with the exception of the absent morning activity, free-running locomotor activity rhythms were astonishingly normal in these flies. This largely contrasts with the weak rhythmicity of the *Pdf^01^* mutants.

### The Role of CCHa1 in Rhythmic Behavior

As already discussed above, PDF and CCHa1, the excitatory neuromessengers of the s-LN_v_ and DN_1a_ neurons, respectively, are necessary for a pronounced morning activity of the flies. In contrast, the two inhibitory neuromessengers of these clock neurons, glutamate and glycine, are not needed for morning activity (Hamasaka et al., 2007; Collins et al., 2014; Frenkel et al., 2017). Evidently, glutamate and CCHa1 from the DN_1a_ neurons affect activity differently. This also holds true for the activity level. Whereas the knockdown of the glutamate receptor increases general activity levels (Collins et al., 2014), we found here that the knockdown of *CCHa1* decreases activity levels. Thus, glutamate inhibits activity, whereas CCHa1 promotes activity, most likely by affecting PDF release. Moreover, CCHa1 does not only promote activity and consequently shorten the siesta; it also changes the phase of activity. Under LD, the *CCHa1* knockdown advances the end of morning activity, whereas under DD conditions, it delays the median of activity. These phasic changes cannot only be detected in the activity pattern, but are even more prominent in the PDP1 oscillations in the s-LN_v_ neurons. The knockdown of the glutamate receptor again exerts different effects on the phase of activity than the knockdown of *CCHa1*: it delays the end of evening activity under LD and DD conditions and consequently delays the onset of night sleep (Hamasaka et al., 2007; Collins et al., 2014). In these studies, the molecular oscillations in the different neurons have not been determined. Therefore, we cannot judge their phase. Nevertheless, in summary, both neuromessengers may cooperate in adjusting the phase of the s-LN_v_ neurons, and consequently, the phase of the activity rhythm.

Interestingly, CCHa1 appears to exert only minor effects on the free-running period of the activity rhythm under DD. This is different for glutamate and glycine, which seem to shorten the period (Hamasaka et al., 2007; Frenkel et al., 2017), and for PDF, which lengthens the period (Renn et al., 1999). We found that the *CCHa1* knockdown shortens the period, implying that CCHa1 might exert a lengthening effect on period, similar to PDF. However, the period of the *CCHa1* mutant was not significantly shorter than that of the controls, suggesting that the effects of CCHa1 on the period are rather small and depend on the genetic background.

### Comparison With the Mammalian Circadian System

The mammalian SCN also uses several neurotransmitters for intercellular coupling, among which VIP and its receptor, VPAC2R, are proposed to have functional homology with the *Drosophila* PDF/PDF-R (Mertens et al., 2005). *Vipr^-/-^* transgenic mice, which lack VPAC2R, show arrhythmic or a shorter free-running rhythm in behavior under constant conditions, which is a phenocopy of the *Pdf* or *Pdf-r Drosophila* mutants (Aton et al., 2005). The mammalian homolog of *CCHa1-R* is the receptor of GRP, bombesin 2 [Flybase, the DRSC Integrative Ortholog Prediction Tool (Hu et al., 2011)], although there is no homolog of *CCHa1*. The GRP-receptor deficient mice show normal free-running activity rhythms in DD but attenuated response to a light-pulse (Aida et al., 2002). The activated GRP-receptor is coupled to Gq protein, which activates phospholipase C and increases Ca^2+^ levels but does not directly elevate cAMP levels (Roesler and Schwartsmann, 2012). Here, we found an increase in cAMP levels in the s-LN_v_, indicating that CCHa1-R couples to a different G protein (**Figure 4**). However, we cannot exclude the possibility that CCHa1-R in the l-LN_v_ functions via a phospholipase C-activating Gq protein that increases Ca^2+^ levels, perhaps in cooperation with light (see also discussion above). If true, this may explain why the l-LN_v_ do not respond to CCHa1 application with an increase of cAMP levels, although they express CCHa1-R (**Figure 3**). If the l-LN_v_ neurons need light and CCHa1 in order to respond—and, in fact, they are light-responsive (Sheeba et al., 2008; Fogle et al., 2011)—this might also explain why the flies respond differently to *CCHa1* knockdown in LD and DD. In LD, they phase advance activity, perhaps because the l-LN_v_ and s-LN_v_ neurons are affected by the absence of CCHa1 and the l-LN_v_ signal on the s-LN_v_ neurons, as has been shown earlier (Yoshii et al., 2009b). In DD, the flies phase delay activity, perhaps because only the s-LN_v_ are affected. Certainly, this hypothesis is highly speculative and needs to be verified by future studies.

Recently, Goda et al. (2018) showed that the calcitonin receptor in mammals and its *Drosophila* homolog, DH31-receptor, share a similar function: controlling circadian body temperature rhythms. Thus, neuropeptide ligands responsible for circadian circuits are not conserved but their G-protein coupled receptors are well-conserved across animal clocks.

## Conclusion

We have shown that CCHa1 signals from the DN_1a_ neurons modulate PDP1 and PDF cycling in the s-LN_v_ neurons, which contribute to a normal morning activity peak. The s-LN_v_ neurons are known as the master pacemaker neurons (Yoshii et al., 2012). However, we conclude that the s-LN_v_ neurons are not the solitary head of the hierarchy; rather, they seem to act in conjunction with the DN_1a_ (CCHa1 and glutamate) and DN_1p_ (glutamate) neurons to control the phase and level of morning activity (Collins et al., 2012, 2014; Guo et al., 2016).

## Author Contributions

YF, CH-L, MK, MS, CH-F, and TY performed and analyzed the experiments. TI generated CCHa1 antibodies and peptide. CH-L performed the cAMP imaging and improved the manuscript. TY and CH-F wrote the manuscript.

## Conflict of Interest Statement

The authors declare that the research was conducted in the absence of any commercial or financial relationships that could be construed as a potential conflict of interest.

## Footnotes

1 http://actogramj.neurofly.de/
