# Supplementary material for: The CCHamide1 Neuropeptide Expressed in the Anterior Dorsal Neuron 1 Conveys a Circadian Signal to the Ventral Lateral Neurons in Drosophila melanogaster
Source: Front Physiol. 2018 Sep 10;9:1276. doi: 10.3389/fphys.2018.01276 (PMC6139358; doi:10.3389/fphys.2018.01276)
Supplement: Supplementary file 1 [file Data_Sheet_1.docx]

Supplementary Material

The CCHamide1 neuropeptide expressed in the anterior dorsal neuron 1 conveys a circadian signal to the ventral lateral neurons in *Drosophila melanogaster*

Yuri Fujiwara, Christiane Hermann-Luibl, Maki Katsura, Manabu Sekiguchi, Takanori Ida, Charlotte Helfrich-Förster*, Taishi Yoshii*

***Correspondence:** Taishi Yoshii: yoshii@okayama-u.ac.jp

***Correspondence:** Charlotte Helfrich-Förster: charlotte.foerster@biozentrum.uni-wuerzburg.de


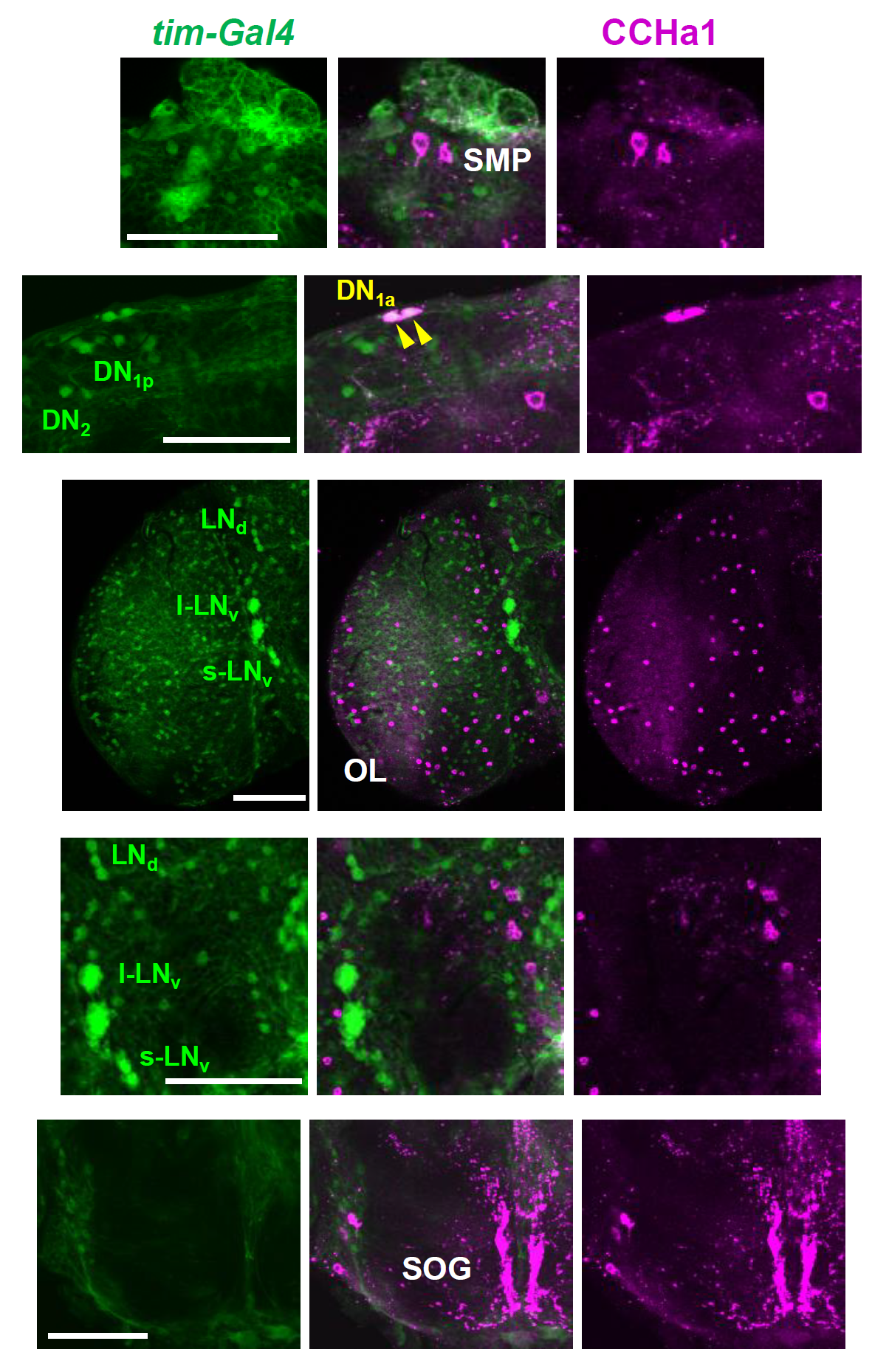


**Supplementary Figure S1.** CCHa1 expression in the DN_1a_ neurons

The brains of *tim-Gal4/UAS-GFP S65T* flies were immunostained with anti-GFP (green) and anti-CCHa1 (magenta) antibodies; CCHa1 and *tim-Gal4* are co-expressed only in the DN_1a_ neurons. OL, optic lobe; SMP, superior medial protocerebrum; SOG, suboesophageal ganglion

**
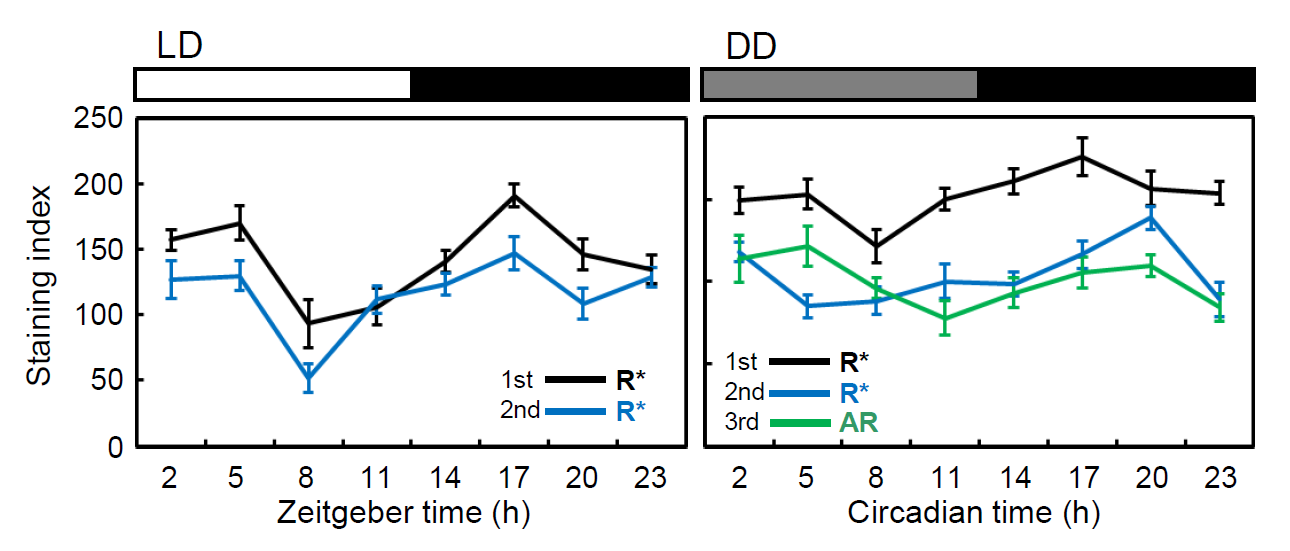
**

**Supplementary Figure S2.** Rhythmic expression of CCHa1 in LD and DD

Mean CCHa1 staining intensity (± SEM) from individual experiments in LD (left) and DD (right). The pooled data are shown in Figure 2. Two (black and blue) and three independent experiments (black, blue, and green) were conducted in LD and DD, respectively. The rhythmicity of CCHa1 expression is indicated by R* (rhythmic) or AR (arrhythmic), analyzed using CircWave (p<0.01) and one-way ANOVA (p<0.05).


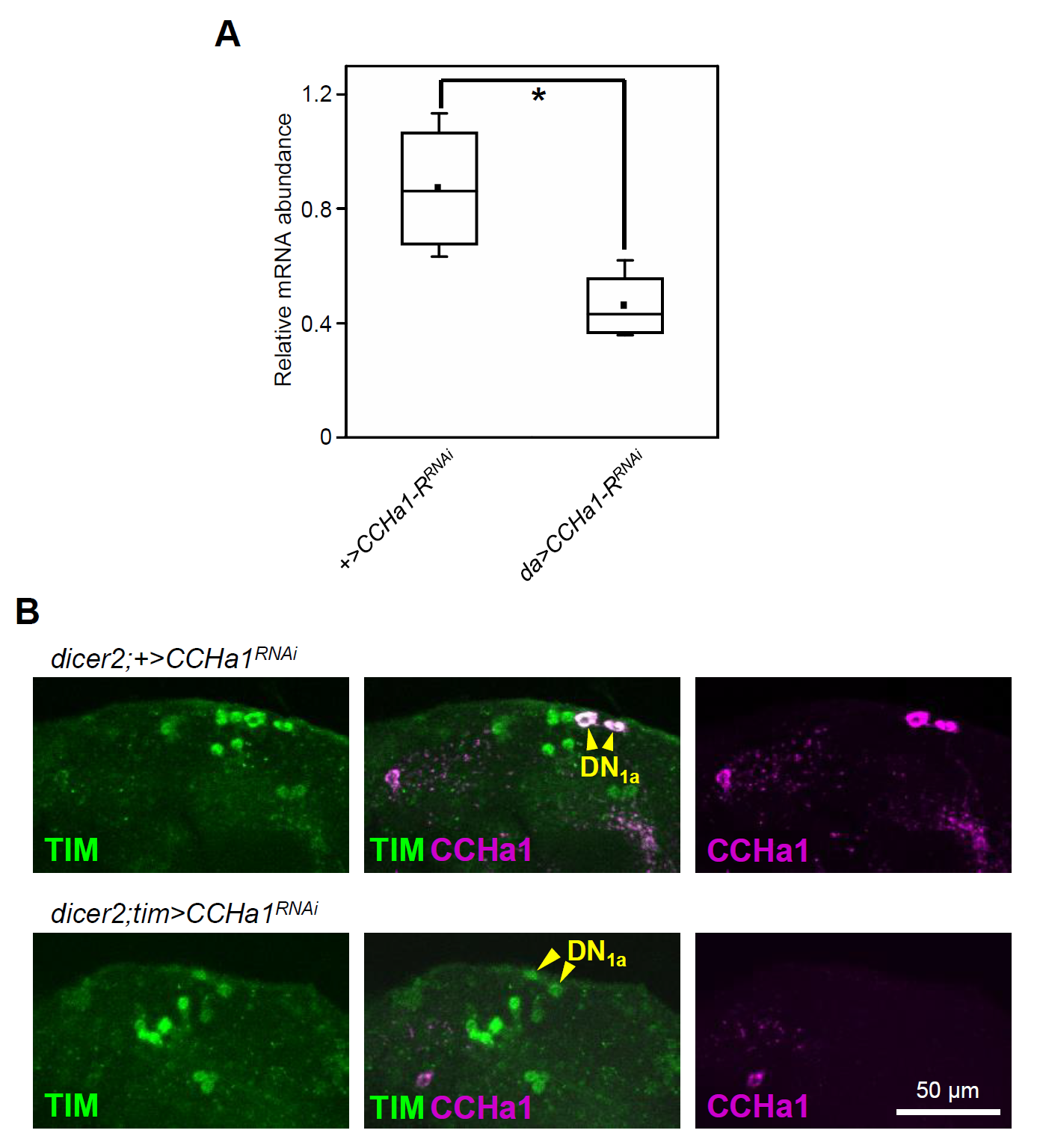


**Supplementary Figure S3.** Validations of *CCHa1-R* and *CCHa1* knockdowns

(A) Quantitative PCR assay to verify the efficiency of *CCHa1-R* RNAi. The *dicer2;+>CCHa1-R^RNAi^* (control) and *dicer2;da>CCHa1-R^RNAi^* strains were sampled at ZT2. The experiments were repeated 4 times. The *CCHa1-R* mRNA level in the *CCHa1-R* knockdown strain was significantly reduced compared with the control strain (p<0.05, Mann-Whitney U test). (B) To visualize clock neurons, anti-TIM antibody (green) was used. In the control strain, CCHa1 and TIM were co-labeled in the DN_1a_ neurons, whereas the CCHa1 staining in the DN_1a_ neurons was completely abolished in *CCHa1* knockdown flies.
